# Supplementary material for: A UMLS-based spell checker for natural language processing in vaccine safety
Source: BMC Med Inform Decis Mak. 2007 Feb 12;7:3. doi: 10.1186/1472-6947-7-3 (PMC1805499; doi:10.1186/1472-6947-7-3)
Supplement: Additional file 13 — RAP application directory. Sets up directory for RAP (RDF application for PHP) source code [file 1472-6947-7-3-S13.gz › rap/doc/phpdoc/default/_constants_php.html]

xml version="1.0" encoding="iso-8859-1"?


Docs for page constants.php


## /constants.php

Description

Description |
Constants

- filesource: Source Code for this file

Constants

Description |
Constants

ADODB\_DB\_DRIVER = 'ODBC'
(line 291)

ADODB\_DB\_HOST = 'rap'
(line 292)

ADODB\_DB\_NAME = ''
(line 293)

ADODB\_DB\_PASSWORD = ''
(line 295)

ADODB\_DB\_USER = ''
(line 294)

ADODB\_DEBUG\_MODE = '0'
(line 296)

ATOM\_NS = 'http://purl.org/atom/ns#'
(line 316)

BNODE\_PREFIX = 'bNode'
(line 64)

CREATE\_MODEL\_WITHOUT\_DUPLICATES = FALSE
(line 104)

DATATYPE\_SHORTCUT\_PREFIX = 'datatype:'
(line 269)

DBMODEL = 'DbModel'
(line 83)

DCMITYPE\_NS = 'http://purl.org/dc/dcmitype/'
(line 319)

DCTERM\_NS = 'http://purl.org/dc/terms/'
(line 318)

DC\_NS = 'http://purl.org/dc/elements/1.1/'
(line 317)

DEFAULT\_ALGORITHM = 'MD5'
(line 39)

DEFAULT\_ENCODING = 'UTF-8'
(line 40)

FIX\_BLANKNODES = TRUE
(line 108)

FOAF\_NS = 'http://xmlns.com/foaf/0.1/#'
(line 320)

GENERAL\_PREFIX\_BASE = 'ns'
(line 176)

HIDE\_ADVERTISE = FALSE
(line 140)

HTML\_TABLE\_BNODE\_COLOR = '#FFCCFF'
(line 187)

HTML\_TABLE\_HEADER\_COLOR = '#FFFFFF'
(line 184)

HTML\_TABLE\_LITERAL\_COLOR = '#E7E7EF'
(line 186)

HTML\_TABLE\_NS\_ROW\_COLOR0 = '#E7E7EF'
(line 190)

HTML\_TABLE\_NS\_ROW\_COLOR1 = '#FFFFFF'
(line 189)

HTML\_TABLE\_RDF\_NS\_COLOR = '#CCFFCC'
(line 188)

HTML\_TABLE\_RESOURCE\_COLOR = '#FFFFCC'
(line 185)

INDENTATION = ' '
(line 41)

INDEX\_TYPE = IND\_DEF
(line 76)

IND\_DEF =
(line 72)

IND\_SO = 3
(line 75)

IND\_SP = 2
(line 74)

IND\_SPO = 1
(line 73)

INFMODELB = 'InfModelB'
(line 85)

INFMODELF = 'InfModelF'
(line 84)

INF\_RES\_DOMAIN = true
(line 351)

INF\_RES\_OWL\_INVERSEOF = true
(line 355)

INF\_RES\_OWL\_SAMEAS = true
(line 353)

INF\_RES\_RANGE = true
(line 349)

INF\_RES\_RULE\_RDFS6 = false
(line 360)

INF\_RES\_RULE\_RDFS8 = false
(line 361)

INF\_RES\_RULE\_RDFS10 = false
(line 362)

INF\_RES\_RULE\_RDFS12 = false
(line 359)

INF\_RES\_RULE\_RDFS13 = false
(line 363)

INF\_RES\_SUBCLASSOF = true
(line 345)

INF\_RES\_SUBPROPERTYOF = true
(line 347)

IN\_DESCRIPTION = 2
(line 114)

IN\_PROPERTY\_EMPTY\_RESOURCE = 5
(line 117)

IN\_PROPERTY\_LITERAL = 6
(line 118)

IN\_PROPERTY\_PARSE\_TYPE\_COLLECTION = 11
(line 123)

IN\_PROPERTY\_PARSE\_TYPE\_LITERAL = 7
(line 119)

IN\_PROPERTY\_PARSE\_TYPE\_RESOURCE = 8
(line 120)

IN\_PROPERTY\_RESOURCE = 4
(line 116)

IN\_PROPERTY\_UNKNOWN\_OBJECT = 3
(line 115)

IN\_RDF = 1
(line 113)

IN\_TOP\_LEVEL =
(line 112)

IN\_UNKNOWN = 10
(line 122)

IN\_XML = 9
(line 121)

LINEFEED = chr(10)
(line 42)

MAX\_ALLOWED\_ABBREVIATED\_LENGTH = 60
(line 177)

MEMMODEL = 'MemModel'
(line 82)

N3SER\_BNODE\_SHORT = FALSE
(line 169)

NAMESPACE\_SEPARATOR\_CHAR = '^'
(line 110)

NAMESPACE\_SEPARATOR\_STRING = '^'
(line 111)

NO\_INDEX = -1
(line 71)

ONTMODEL = 'OntModel'
(line 86)

OWL\_INVERSE\_OF = 'inverseOf'
(line 258)

OWL\_NS = 'http://www.w3.org/2002/07/owl#'
(line 321)

OWL\_PREFIX = 'owl'
(line 256)

OWL\_SAME\_AS = 'sameAs'
(line 257)

OWL\_URI = 'http://www.w3.org/2002/07/owl#'
(line 255)

PACKAGE\_DBASE = 'model/DBase.php'
(line 49)

PACKAGE\_INFMODEL = 'infModel/InfModelP.php'
(line 55)

PACKAGE\_MODEL = 'model/ModelP.php'
(line 47)

PACKAGE\_ONTMODEL = 'ontModel/OntModelP.php'
(line 57)

PACKAGE\_RDQL = 'rdql/RDQL.php'
(line 54)

PACKAGE\_RESMODEL = 'resModel/ResModelP.php'
(line 56)

PACKAGE\_SYNTAX\_GRDDL = 'syntax/SyntaxGRDDL.php'
(line 52)

PACKAGE\_SYNTAX\_N3 = 'syntax/SyntaxN3.php'
(line 51)

PACKAGE\_SYNTAX\_RDF = 'syntax/SyntaxRDF.php'
(line 50)

PACKAGE\_UTILITY = 'util/Utility.php'
(line 48)

PACKAGE\_VOCABULARY = 'vocabulary/Vocabulary.php'
(line 53)

RDFAPI\_ERROR = 'RDFAPI error '
(line 38)

RDFS\_CLASS = 'Class'
(line 241)

RDFS\_COMMENT = 'comment'
(line 248)

RDFS\_DATATYPE = 'Datatype'
(line 243)

RDFS\_DOMAIN = 'domain'
(line 240)

RDFS\_IS\_DEFINED\_BY = 'isDefinedBy'
(line 246)

RDFS\_LABEL = 'label'
(line 247)

RDFS\_LITERAL = 'Literal'
(line 244)

RDFS\_RANGE = 'range'
(line 239)

RDFS\_RESOURCE = 'Resource'
(line 242)

RDFS\_SEE\_ALSO = 'seeAlso'
(line 245)

RDFS\_SUBCLASSOF = 'subClassOf'
(line 237)

RDFS\_SUBPROPERTYOF = 'subPropertyOf'
(line 238)

RDFS\_VOCABULARY = 'RdfsVocabulary.php'
(line 88)

RDF\_ABOUT = 'about'
(line 201)

RDF\_ABOUT\_EACH = 'aboutEach'
(line 202)

RDF\_ABOUT\_EACH\_PREFIX = 'aboutEachPrefix'
(line 203)

RDF\_ALT = 'Alt'
(line 214)

RDF\_BAG = 'Bag'
(line 212)

RDF\_BAG\_ID = 'bagID'
(line 204)

RDF\_DATATYPE = 'datatype'
(line 221)

RDF\_DATATYPE\_SCHEMA\_URI = 'http://www.w3.org/TR/xmlschema-2'
(line 235)

RDF\_DESCRIPTION = 'Description'
(line 199)

RDF\_FIRST = 'first'
(line 227)

RDF\_ID = 'ID'
(line 200)

RDF\_LI = 'li'
(line 215)

RDF\_LIST = 'List'
(line 224)

RDF\_NAMESPACE\_PREFIX = 'rdf'
(line 197)

RDF\_NAMESPACE\_URI = 'http://www.w3.org/1999/02/22-rdf-syntax-ns#'
(line 196)

RDF\_NIL = 'nil'
(line 225)

RDF\_NODEID = 'nodeID'
(line 220)

RDF\_OBJECT = 'object'
(line 219)

RDF\_OBJECT\_TYPE\_BNODE = 3
(line 132)

RDF\_OBJECT\_TYPE\_LITERAL = 1
(line 130)

RDF\_OBJECT\_TYPE\_RESOURCE =
(line 129)

RDF\_OBJECT\_TYPE\_XML = 2
(line 131)

RDF\_PARSE\_TYPE = 'parseType'
(line 207)

RDF\_PARSE\_TYPE\_COLLECTION = 'Collection'
(line 210)

RDF\_PARSE\_TYPE\_LITERAL = 'Literal'
(line 208)

RDF\_PARSE\_TYPE\_RESOURCE = 'Resource'
(line 209)

RDF\_PREDICATE = 'predicate'
(line 218)

RDF\_PROPERTY = 'Property'
(line 223)

RDF\_RDF = 'RDF'
(line 198)

RDF\_RESOURCE = 'resource'
(line 205)

RDF\_REST = 'rest'
(line 226)

RDF\_SCHEMA\_PREFIX = 'rdfs'
(line 236)

RDF\_SCHEMA\_URI = 'http://www.w3.org/2000/01/rdf-schema#'
(line 234)

RDF\_SEEALSO = 'seeAlso'
(line 222)

RDF\_SEQ = 'Seq'
(line 213)

RDF\_STATEMENT = 'Statement'
(line 216)

RDF\_SUBJECT = 'subject'
(line 217)

RDF\_SUBJECT\_TYPE\_ANONYMOUS = 3
(line 127)

RDF\_SUBJECT\_TYPE\_BNODE = 4
(line 128)

RDF\_SUBJECT\_TYPE\_DISTRIBUTED = 1
(line 125)

RDF\_SUBJECT\_TYPE\_PREFIX = 2
(line 126)

RDF\_SUBJECT\_TYPE\_URI =
(line 124)

RDF\_TYPE = 'type'
(line 211)

RDF\_VALUE = 'value'
(line 206)

RDF\_XMLLITERAL = 'XMLLiteral'
(line 228)

RDQL\_AND\_ERR = RDQL\_ERR.'in the AND clause: '
(line 308)

RDQL\_ERR = 'RDQL error '
(line 303)

RDQL\_SEL\_ERR = RDQL\_ERR.'in the SELECT clause: '
(line 305)

RDQL\_SRC\_ERR = RDQL\_ERR.'in the SOURCE clause: '
(line 306)

RDQL\_SYN\_ERR = 'RDQL syntax error '
(line 304)

RDQL\_USG\_ERR = RDQL\_ERR.'in the USING clause: '
(line 309)

RDQL\_WHR\_ERR = RDQL\_ERR.'in the WHERE clause: '
(line 307)

RESMODEL = 'ResModel'
(line 87)

RSS\_NS = 'http://purl.org/rss/1.0/#'
(line 322)

SER\_RDF\_QNAMES = TRUE
(line 161)

SER\_SORT\_MODEL = FALSE
(line 151)

SER\_USE\_ATTRIBUTES = FALSE
(line 147)

SER\_USE\_ENTITIES = FALSE
(line 143)

SER\_XML\_DECLARATION = TRUE
(line 165)

UNIC\_RDF = TRUE
(line 100)

USE\_ANY\_QUOTE = FALSE
(line 175)

USE\_CDATA = FALSE
(line 173)

VALIDATE\_IDS = TRUE
(line 96)

VCARD\_NS = 'http://www.w3.org/2001/vcard-rdf/3.0#'
(line 323)

XML\_LANG = 'lang'
(line 268)

XML\_NAMESPACE\_DECLARATION\_PREFIX = 'xmlns'
(line 266)

XML\_NAMESPACE\_PREFIX = 'xml'
(line 265)

XML\_NAMESPACE\_URI = 'http://www.w3.org/XML/1998/namespace'
(line 267)

Documentation generated on Fri, 17 Dec 2004 16:14:25 +0100 by phpDocumentor 1.3.0RC3
